# Genome-Wide Association Scan Shows Genetic Variants in the *FTO* Gene Are Associated with Obesity-Related Traits

**DOI:** 10.1371/journal.pgen.0030115

**Published:** 2007-07-20

**Authors:** Angelo Scuteri, Serena Sanna, Wei-Min Chen, Manuela Uda, Giuseppe Albai, James Strait, Samer Najjar, Ramaiah Nagaraja, Marco Orrú, Gianluca Usala, Mariano Dei, Sandra Lai, Andrea Maschio, Fabio Busonero, Antonella Mulas, Georg B Ehret, Ashley A Fink, Alan B Weder, Richard S Cooper, Pilar Galan, Aravinda Chakravarti, David Schlessinger, Antonio Cao, Edward Lakatta, Gonçalo R Abecasis

**Affiliations:** 1 Unità Operativa Geriatria, Istituto per la Patologia Endocrina e Metabolica, Rome, Italy; 2 Gerontology Research Center, National Institute on Aging, Baltimore, Maryland, United States of America; 3 Center for Statistical Genetics, Department of Biostatistics, University of Michigan, Ann Arbor, Michigan, United States of America; 4 Istituto di Neurogenetica e Neurofarmacologia, Consiglio Nazionale delle Ricerche, Cittadella Universitaria di Monserrato, Monserrato, Cagliari, Italy; 5 Unità Operativa Semplice Cardiologia, Divisione di Medicina, Presidio Ospedaliero Santa Barbara, Iglesias, Italy; 6 Institute of Genetic Medicine, Johns Hopkins University School of Medicine, Baltimore, Maryland, United States of America; 7 Department of Internal Medicine, University of Michigan School of Medicine, Ann Arbor, Michigan, United States of America; 8 Department of Preventive Medicine and Epidemiology, Loyola Stritch School of Medicine, Chicago, Illinois, United States of America; 9 Institut Scientifique et Technique de la Nutrition et de l'Alimentation, Paris, France; 10 INSERM, U557 (UMR INSERM/INRA/CNAM), Paris, France; Stanford University School of Medicine, United States of America

## Abstract

The obesity epidemic is responsible for a substantial economic burden in developed countries and is a major risk factor for type 2 diabetes and cardiovascular disease. The disease is the result not only of several environmental risk factors, but also of genetic predisposition. To take advantage of recent advances in gene-mapping technology, we executed a genome-wide association scan to identify genetic variants associated with obesity-related quantitative traits in the genetically isolated population of Sardinia. Initial analysis suggested that several SNPs in the *FTO* and *PFKP* genes were associated with increased BMI, hip circumference, and weight. Within the *FTO* gene, rs9930506 showed the strongest association with BMI (*p =* 8.6 ×10*^−^* ^7^), hip circumference (*p =* 3.4 × 10*^−^* ^8^), and weight (*p =* 9.1 × 10*^−^* ^7^). In Sardinia, homozygotes for the rare “G” allele of this SNP (minor allele frequency = 0.46) were 1.3 BMI units heavier than homozygotes for the common “A” allele. Within the PFKP gene, rs6602024 showed very strong association with BMI (*p =* 4.9 × 10*^−^* ^6^). Homozygotes for the rare “A” allele of this SNP (minor allele frequency = 0.12) were 1.8 BMI units heavier than homozygotes for the common “G” allele. To replicate our findings, we genotyped these two SNPs in the GenNet study. In European Americans (*N* = 1,496) and in Hispanic Americans (*N* = 839), we replicated significant association between rs9930506 in the FTO gene and BMI (*p*-value for meta-analysis of European American and Hispanic American follow-up samples, *p =* 0.001), weight (*p =* 0.001), and hip circumference (*p =* 0.0005). We did not replicate association between rs6602024 and obesity-related traits in the GenNet sample, although we found that in European Americans, Hispanic Americans, and African Americans, homozygotes for the rare “A” allele were, on average, 1.0–3.0 BMI units heavier than homozygotes for the more common “G” allele. In summary, we have completed a whole genome–association scan for three obesity-related quantitative traits and report that common genetic variants in the *FTO* gene are associated with substantial changes in BMI, hip circumference, and body weight. These changes could have a significant impact on the risk of obesity-related morbidity in the general population.

## Author Summary

Although twin and family studies have clearly shown that genes play a role in obesity, it has proven quite difficult to identify the specific genetic variants involved. Here, we take advantage of recent technical and methodological advances to examine the role of common genetic variants on several obesity-related traits. By examining >4,000 Sardinians, we show that a specific genetic variant, rs9930506, and other nearby variants on human Chromosome 16 are associated with body mass index, hip circumference, and total body weight. The variants overlap *FTO,* a gene with poorly understood function. Further studies of the region may implicate new biological pathways affecting susceptibility to obesity. We also show that the association is not restricted to Sardinia but is also seen in independent samples of European Americans and Hispanic Americans. This finding is particularly important because obesity is associated with increased risk of cardiovascular disease and diabetes.

## Introduction

There is a worldwide epidemic of obesity and type 2 diabetes across all age groups, especially in industrialized countries [1]. In the United States alone, over two-thirds of the population has a body mass index (BMI) of 25 kg/m^2^ or greater and is thus overweight [2,3]. Being overweight is a well-established risk factor for many chronic diseases, such as type 2 diabetes, hypertension, and cardiovascular events [4], and increases in BMI are associated with higher all-cause mortality [5,6]. The economic cost attributable to obesity in the United States has been estimated to be as high as $100 billion/yr [7], and includes not only direct health care costs but also the cost of lost productivity in affected individuals [8].

Individual susceptibility to obesity is thought to be determined by interactions between an individual's genetic make-up and behavior and the environment. Thus, the increased prevalence of obesity likely reflects the exposure of genetically susceptible individuals to unhealthy secular trends in environmental and behavioral factors, such as diet and exercise [9]. In industrialized countries, between 60%–70% of the variation in obesity-related phenotypes appears to be heritable [10,11].

The traditional approach for mapping disease genes relies on linkage mapping followed by progressive fine-mapping of candidate linkage peaks [12]. While the approach has been extremely successful at identifying genes that predispose carriers to rare Mendelian disorders [13], it has met only limited success when applied to complex traits such as obesity. We have taken advantage of recent advances in genotyping technology that enable detailed assessment of entire genomes [14,15]. These advances have already allowed the identification of genes that influence quantitative variation in heart disease–related phenotypes [16] and of susceptibility genes for age-related macular degeneration [17], inflammatory bowel disease [18], and type 2 diabetes [19].

We recruited and phenotyped 6,148 individuals, male and female, ages 14–102 y, from a cluster of four towns in the Lanusei Valley in the Sardinian province of Ogliastra [20]. By studying an isolated population, we expected to increase the genetic and environmental homogeneity of our sample, increasing power [21,22]. Our cohort included >30,000 relative pairs and represents >60% of the population eligible for participation in the study; a detailed account of the family structures we examined is available elsewhere [20]. We took advantage of the relatedness among individuals in our sample to substantially reduce study costs [23]. Specifically, because our sample includes many large families, we reasoned that genotyping a relatively small number of markers in all individuals would allow us to identify shared haplotype stretches within each family. We could then genotype a subset of the individuals in each family at higher density to characterize the haplotypes in each stretch and impute missing genotypes in other individuals in the family [23,24].

For the analyses presented here, we genotyped 3,329 individuals using the Affymetrix 10,000 SNP Mapping Array and we genotyped an additional 1,412 individuals using the Affymetrix 500,000 SNP Mapping Array Set. The genotyped individuals were selected to represent the largest families in our sample, without respect to phenotype. The high-density arrays were generally used to genotype both parents and one child (in larger sibships) or just the parents (in smaller sibships); the lower density arrays were used to genotype everyone else. Except when parents and offspring were genotyped in the same family, we tried to ensure that individuals genotyped with the high-density array were only distantly related to one another. For the 2,893 individuals that were genotyped with the 10,000 SNP arrays only, we used a modified version of the Lander-Green algorithm [25,26] to probabilistically infer missing genotypes [24]. Our approach for estimating missing genotypes is implemented in MERLIN (http://www.sph.umich.edu/csg/abecasis/MERLIN/) and described in detail elsewhere [24]. Our initial analysis focused on evaluating the additive effects of 362,129 SNPs (Table S1) that passed quality control checks [27,28]. The remaining SNPs failed quality checks (∼2.9% of SNPs failed checks for data completeness, Hardy–Weinberg equilibrium, and Mendelian incompatibilities) or had a minor allele frequency of <5% (∼25.7% of SNPs had low minor allele frequencies).

## Results

We tested 362,129 SNPs for association with three obesity-related quantitative traits (BMI, hip circumference, and weight). Height was included as a covariate in analysis of hip circumference and weight. In addition, we included age and sex as covariates in every analysis. The genomic control parameter [29] for our initial analysis of each trait ranged from 1.07 to 1.09, indicating that our estimated test statistics might be slightly inflated. This is likely due to unaccounted-for distant relationships among the sampled individuals. All results presented in our tables have been adjusted using the method of genomic control [29]. After adjustment, we observed no significant excess of results exceeding liberal significance thresholds. For example, the proportion of test statistics that were significant at α = 0.001 was 0.00098.

**Figure 1:**
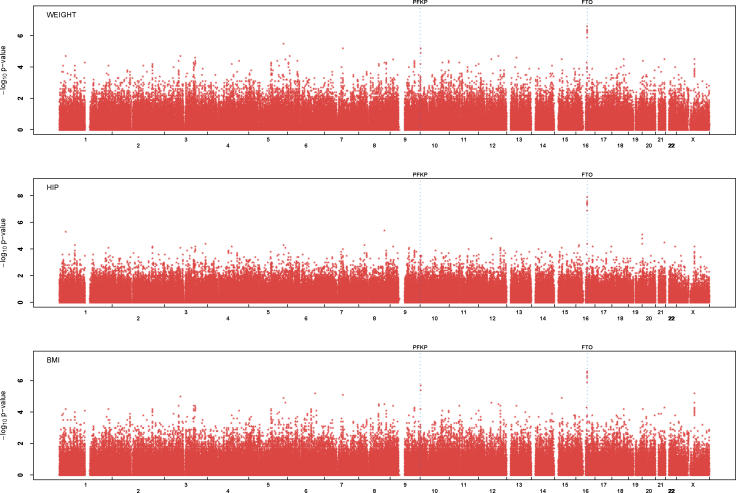
Negative Log of *p*-Value for Single Marker Association Analysis with Three Obesity-Related Traits Locations of *PFKP* and *FTO* genes are highlighted.

Results of our initial association analysis are summarized in Figure 1 and in Table 1. We used the false-discovery rate (FDR) to select a small set of very promising trait SNP associations for rapid replication. Using an FDR [30] of 20% highlighted a small set of SNPs for each trait. This set include the top eight SNP association results for hip circumference and weight (FDR = 0.013 and FDR = 0.16, respectively) and the top nine SNP association results for BMI (FDR = 0.20).

**Table 1:**
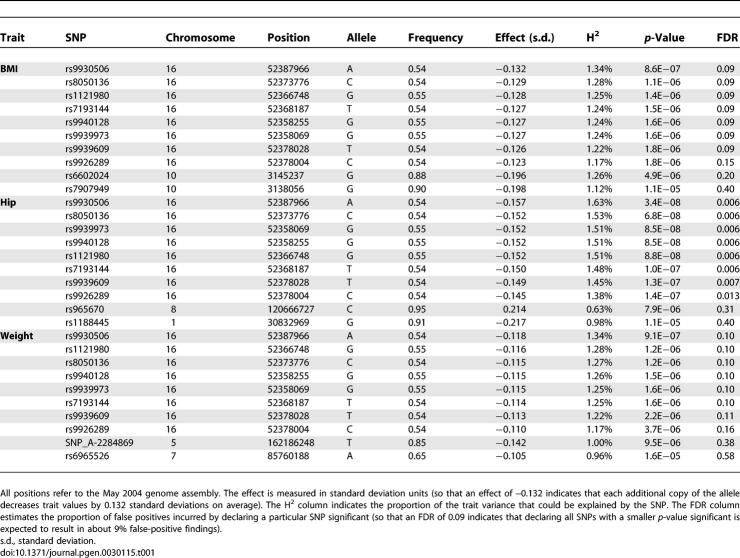
Markers Showing Strongest Evidence for Association

Eight of the SNPs listed in Table 1 overlap among the three traits. In particular, SNP rs9930506 and a cluster of nearby SNPs on Chromosome 16 show strong association with BMI (*p =* 8.6 × 10*^−^* ^7^), hip circumference (*p =* 3.4 × 10*^−^* ^8^) and weight (*p =* 9.1 × 10*^−^* ^7^). Two of the associated SNPs in the cluster, rs9939609 and rs9926289, fall within an intronic region where sequence is strongly conserved across species. For comparative purposes, using a conservative Bonferroni correction aimed at an overall type I error rate of 0.05 (one false positive per 20 genome-scans), would result in a significance threshold of 1.4 × 10*^−^* ^7^.

**Figure 2:**
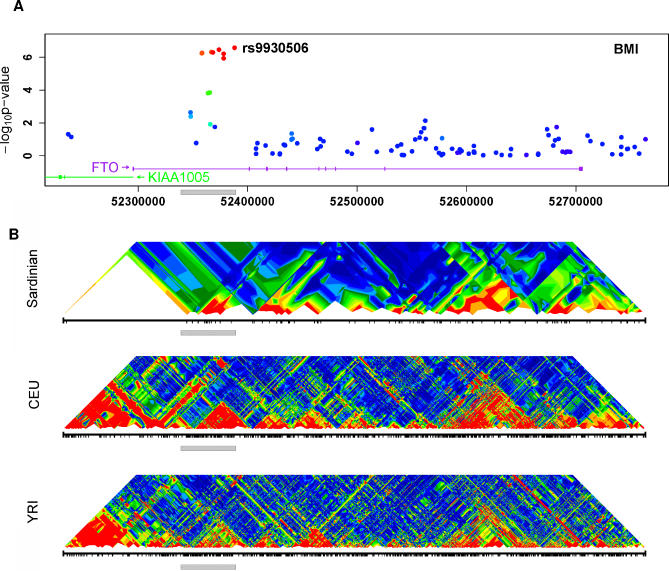
Association Results and LD Patterns in Region Surrounding the *FTO* Gene (A) Summary of the association between SNPs in the region and BMI. The SNP showing strongest association (rs9930506) is highlighted. Other SNPs are colored according to their degree of disequilibrium with rs9930506 ranging from high (red), to intermediate (green), to low (blue). Transcripts are indicated at the bottom of the graph, with an arrow indicating transcript direction. (B) Summary of the patterns of disequilibrium in the region in Sardinia and in two of the HapMap populations (CEU and YRI) [55]. The grey bar marks the region of association and facilitates comparisons between the panels.

This cluster of SNPs on Chromosome 16 overlaps the *FTO* [31] gene, an extremely large gene whose exons span >400kb (Figure 2). KIAA1005, a gene of unknown function, also maps nearby. The *FTO* gene has not been previously implicated in obesity, but it maps to a region where linkage to BMI has been reported in two previous genome-wide linkage scans (LOD = 3.2 in the Framingham Heart Study [32] and LOD = 2.2 in the families with white ancestry from the Family Blood Pressure Program [33]). Furthermore, a syndrome that results from deletion of this region of Chromosome 16q includes obesity as one of its features [34].

Although multiple SNPs within *FTO* show evidence for association, these do not point to multiple independently associated SNPs—rather, it is likely they are all in disequilibrium with the same causal variant(s). In a sequential analysis in which we selected the best SNP for each trait and then conditioned on it to successively select the next best SNP, only one *FTO* SNP was selected (results presented in Table S2). This result is consistent with the fact that the SNPs fall in a region of strong linkage disequilibrium, both in Sardinia and in the HapMap (Figure 2B).

**Figure 3:**
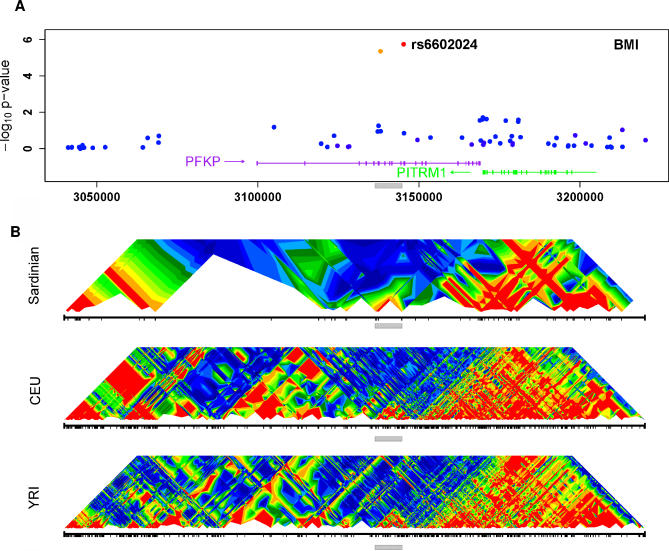
Association Results and LD Patterns in Region Surrounding the *PFKP* Gene (A) Summary of the association between SNPs in the region and BMI. The SNP showing strongest association (rs6602024) is highlighted. Other SNPs are colored according to their degree of disequilibrium with rs6602024, ranging from high (orange) to low (blue). Transcripts are indicated at the bottom of the graph, with an arrow indicating transcript direction. (B) Summary of the patterns of disequilibrium in the region in Sardinia and in two of the HapMap populations (CEU and YRI) [55]. The grey bar marks the region of association and facilitates comparisons between the panels.

Our FDR analysis of BMI selected one additional SNP outside this cluster, rs6602024 (Figure 3). This SNP maps to Chromosome 10 and shows association with BMI (*p =* 4.9 × 10*^−^* ^6^), weight (1.6 × 10*^−^* ^5^), and hip circumference (*p =* 0.00047). The SNP maps to the platelet-type phosphofructokinase (*PFKP*) gene, which acts as a major rate-limiting enzyme in glycolysis, converting D-fructose-6-phosphate to fructose-1,6-bisphosphate [35]. Alterations in the structure or regulation of *PFKP* could alter the balance between glycolysis and glycogen production, ultimately leading to obesity.

Table 2 shows the phenotypic effects associated with each of the two SNPs in our sample. Because rs9930506 is more common, it shows more significant association despite being associated with smaller phenotypic effects (the two homozygotes differ, on average, by ∼1.5 BMI units). A rarer polymorphism, such as rs6602024, impacts only a smaller proportion of the population and shows less significant association, despite a larger difference between homozygote means (which differ, on average, by ∼2.9 BMI units). In each case, a more accurate estimate of the effect is provided by the regression model with age, sex, and (where appropriate) height as covariates. In a study, such as ours, that estimates effect sizes for many SNPs, statistical fluctuation means that some estimates will be slightly high and others will be low. SNPs that reach statistical significance are likely to include those for which effect size estimates are inflated (this is the winner's curse phenomenon) [36], and thus we proceeded to replicate our top association signals in additional large samples.

**Table 2:**
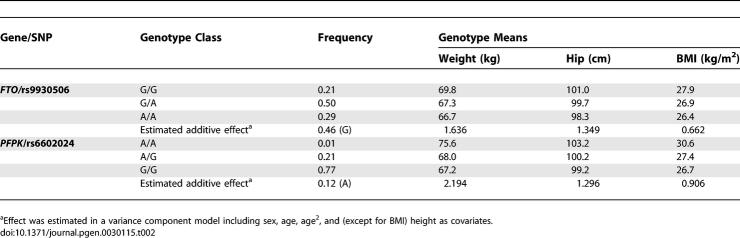
Effects Associated with the rs9930506 and rs6602024 SNPs

To further investigate the association between rs9930506 and rs6602024 and obesity-related traits, we genotyped these SNPs in the GenNet study [37]. The study includes a series of families recruited through probands with elevated blood pressure. The families included in this analysis comprise 3,467 individuals in total (1,101 African Americans [AA] in 369 families, 839 Hispanic Americans [HA] in 223 families, and 1,496 European Americans [EA] in 457 families). Overall, individuals in GenNet are heavier than those in our original Sardinian sample. Nevertheless, our findings strongly confirm evidence for association between rs9930506 and the three BMI-related traits (weight, hip circumference, and BMI). Specifically, rs9930506 showed association with all three traits among EA and HA in the GenNet study (meta-analysis of the EA and HA samples results in a *p*-value between 0.0005 and 0.001, depending on trait; see Table 3). The association is significant and in the same direction as in our original sample. The allele frequencies are also similar in all three samples, with a frequency of 0.46 in our Sardinian sample for allele “G” of rs9930506 and of 0.44 and 0.33 in the GenNet EA and HA samples, respectively. In the GenNet sample, homozygotes for the two rs9930506 alleles differ in weight by ∼1.0 BMI units on average.

**Table 3:**
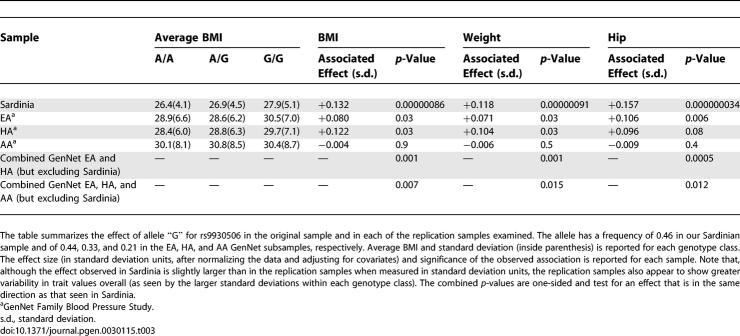
Replication of Association between rs9930506/*FTO*/G Allele and Obesity-Related Traits

We also examined the relationship between rs9930506 and the three traits in AA, but did not observe evidence for association within that group. In AA, allele “G” of marker rs9930506 has a somewhat lower frequency of 0.21. In addition, AA show quite distinct patterns of linkage disequilibrium (LD) and thus it is not surprising that the association does not replicate. For example, in the HapMap sample of Utah residents with ancestry from northern and western Europe (CEU), the eight SNPs that show association with obesity-related traits in our sample are strongly associated with each other and tag a total of 38 different variants (*r* ^2^ > 0.80). In contrast, in the HapMap Yoruba in Ibadan, Nigeria (YRI) the strength of LD in the region is greatly reduced such that rs9930506 is not in strong LD (*r* ^2^ < 0.3) with any of the other Chromosome 16 SNPs that show association in Sardinia.

In an attempt to fine-map association in the region, we decided to genotype the region of strong association in greater detail. In general, the study of samples from AA participants can afford an opportunity to fine-map association signals and even facilitate identification of the causal variants [38]. As noted above, a total of 38 different variants are in LD (*r* ^2^ > 0.8, HapMap CEU) with the eight SNPs that are associated with obesity-related traits in our Sardinian sample. We selected an additional seven SNPs in the region to tag these 38 variants in samples with reduced LD. Together with rs9930506, these seven variants capture the other 30 SNPs with *r* ^2^ > 0.58 (average *r* ^2^ = 0.87, HapMap YRI). The results are summarized in Table 4 and show that, whereas all the variants show association in EA and HA, none of the variants shows association in AA. One possible explanation is that obesity in AA has a different genetic architecture. Alternatively, it is possible that because some of the variants are quite common in EA and HA but rare in AA, much larger sample sizes will be required to adequately gauge their effects (for example, rs1421085 and rs3751812 have minor allele frequencies >0.25 in these first two populations, but <0.11 in AA).

**Table 4:**
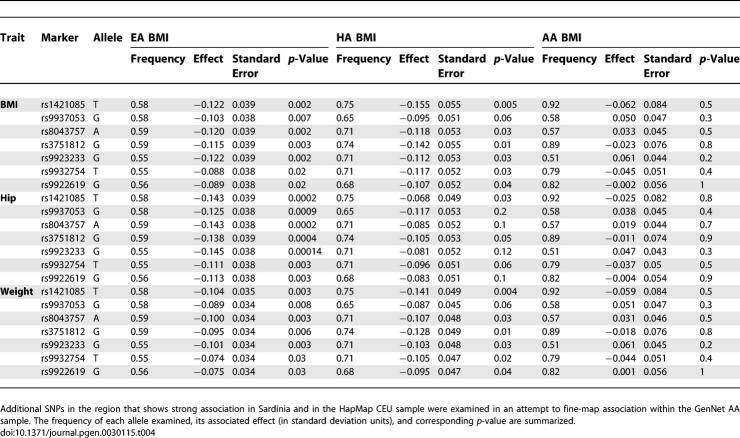
Fine-Mapping Results for *FTO* Region in GenNet Sample

In contrast to rs9930506, we did not replicate association between SNP rs6602024 in the *PFKP* gene and the three obesity-related traits. The “A” allele was rare in all populations, with a frequency of 0.12 in our Sardinian sample, 0.11 in the HA and EA GenNet subsamples and 0.25 in the AA GenNet subsample. The results are summarized in Table 5 and show that, although homozygotes for the rare “A” allele at rs6602024 were on average heavier by ∼1.0–3.0 BMI units than homozygotes for the “G” allele at the SNP, these homozygotes were rare and, overall, there was no significant association. Corroborating evidence that *PFKP* and rs6602024 are associated with BMI is the observation that a region of ∼120 kb including the *Pfkp* gene has been implicated in a mouse model of obesity [39] (see Discussion). A definite assessment of the impact of PFKP on obesity-related quantitative traits in human populations will likely require examination of much larger sample sizes.

**Table 5:**
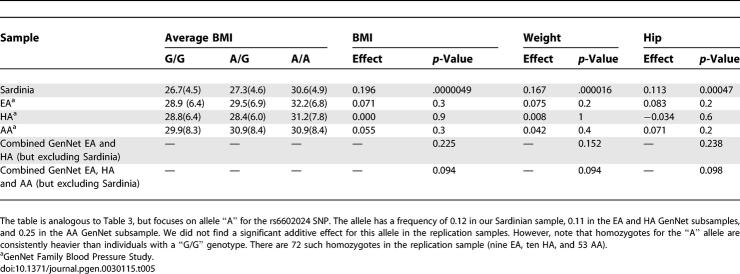
Replication of Association between rs6602024/*PFKP*/A Allele and Obesity-Related Traits

Our genotyping results also hint at the possible importance in Sardinia of other genes previously investigated as candidates influencing obesity and related traits (Tables S3–S5). When we evaluated evidence for association across previously identified candidate genes, we observed a small excess of nominally significant *p*-values. (We tested 837 candidate SNPs in 74 candidate genes against three traits and found that 145 tests were significant at *p* < 0.05, corresponding to 5.8% of the 2,511 tests. We observed no such excess when the whole genome was considered.) Among the interesting candidates that show association in our sample are the two adiponectin receptor genes [40] *ADIPOR1* (best single SNP *p*-value = 0.013, 0.027, and 0.016 for BMI, hip circumference, and weight) and *ADIPOR2* (best *p*-values = 0.018, 0.019, 0.013) and the lipoprotein lipase gene, *LPL* [41] (best *p*-values = 0.014, 0.006, 0.018). Nevertheless, all the association signals observed in any of these previous candidate genes are far less significant than those in *FTO* or *PFKP*.

## Discussion

*FTO* association provides an example of how genome-wide association studies can point to previously unsuspected candidate genes. An interstitial deletion overlapping the region produces human syndromic obesity [34] and a hint that the gene might be involved in stress responses stems from the observation that it is down-regulated when the heat shock response transcription factor *Htf1* is inhibited [42]. Because the gene has no recognizable functional domains and has not been studied in detail in experimental models, no putative function can be currently imputed. The fact that *FTO* is associated not only with BMI but also with hip circumference and weight is consistent with previous analyses of heritability in our cohort [20]. The analyses suggested that 80% of the genetic variance of these traits is determined by common loci (individually, the traits have heritabilities between ∼30%–45%). Although the three traits examined here are correlated (all pairwise correlations were >0.73), it is important to note that apart from the SNPs that overlap *FTO,* other strongly associated SNPs differed among the traits (see Tables 1 and S2).

In contrast to *FTO*, *PFKP* is a critical enzyme within the well-studied pathway of glucose metabolism but, to our knowledge, has not been previously implicated in obesity in humans. PFKP is one of the three phosphofructokinase subunit proteins that show partially overlapping patterns of expression and form hetero-tetramers in diverse cells and tissues. The subunits are encoded by different genes. One form is highly expressed in muscle *(PFKM)*; a second, in liver *(PFKL);* and the third, *PFKP,* is the only form in platelets and is also highly expressed in subregions of the brain [42]. None of the forms has been previously implicated in obesity in humans, although *PFKM* is mutated in some cases of impaired glycogen synthesis (glycogen storage disease VII; see Online Mendelian Inheritance in Man, http://www.ncbi.nlm.nih.gov/entrez/dispomim.cgi?id=232800) [35]. It is of considerable interest that compared to the other isozymes, *PFKP* has lower affinity for fructose-6-phosphate and decreased inhibition by ATP [43]. Consequently, *PFKP* is the most stringently regulated, responding to small changes at typical metabolic levels of effectors [44]. Genetic variants in the enzyme could thus adjust the rate of glycolysis, shifting the balance of metabolism between gluconeogenesis and glucose assimilation—a possible step in the etiology of obesity. Additionally, it is intriguing that in mice a locus associated with obesity has been mapped to a 127-kb interval that includes *Pfkp* [39]. The mouse locus shows strong evidence of interaction with diet, with different effects in mice fed high-fat and low-fat diets. One possibility is that greater homogeneity of diet in Sardinia facilitated mapping, but made replication in other populations more difficult.

How significant are the associations observed? The replication of the *FTO* association in two different populations indicates that it is likely important not only in Sardinia, but in many different populations. In contrast, the failure to replicate the *PFKP* association in other populations suggests that (a) the association we identified may refer to rarer, population-specific variants; (b) the effects of the locus may depend on genetic or environmental background; or (c) the association identified in our original sample is due to the statistical fluctuations inherent in testing hundreds of thousands of SNPs. As for the public health impact of the observed associations, a 1-unit increment in BMI has been associated with an 8% increase in the risk of coronary heart disease [45] and excess weight in middle life is associated with increased overall risk of death [46]. Thus, the alleles reported here, which shift BMI by 1–1.5 units, have effects that are not only statistically significant but could also have important health consequences. Furthermore, apart from the direct contribution of these gene variants, they provide an entrée to the analysis of genes and pathways that contribute additionally, and open new routes to possible eventual intervention.

*Note:* After completing this manuscript, we became aware of additional evidence that supports our report of association between *FTO* and obesity-related traits. First, genotyping of 1,780 individuals from the SUVIMAX study [47,48] replicated association of allele rs9930506 with increased BMI (*p =* 0.006). Combined evidence from SUVIMAX, GenNet EA, and GenNet HA resulted in a replication *p*-value of 1.5 × 10*^−^* ^5^. In addition, two other large independent studies also show association of SNPs in *FTO* with increased BMI [49,50]. Genotyping of the SUVIMAX sample did not provide evidence for association between rs6602024 and BMI.

## Materials and Methods

### Study sample.

We recruited and phenotyped 6,148 individuals, male and female, ages 14–102 y, from a cluster of four towns in the Lanusei Valley [20]. During physical examination of each individual, a blood sample was collected (for DNA extraction) and anthropometric traits were recorded. Here, we report analyses of hip circumference, weight, and the derived quantity BMI (which is calculated from a combination of height and weight). Genotyping was carried out using the Affymetrix 10K and 500K chips (http://affymetrix.com/) using standard protocols. Summary assessments of genotype data quality are provided in the Results section and in Table S1.

To follow up on SNPs rs9930506 and rs6602024, we genotyped and examined the association between these two SNPs and BMI, hip circumference, and body weight in the GenNet study. The study comprises 3,467 individuals in total, recruited between 1995 and 2004 (1,101 AA, 839 HA, and 1,496 EA). Individuals were recruited at two field centers: EA were recruited from Tecumseh, Michigan, and AA and HA were recruited from Maywood, Illinois. Participants were recruited from families starting from a proband with high blood pressure. DNA was available for 3,205 individuals (968 AA, 824 HA, and 1471 EA). SNP genotyping was performed using the 5′-nuclease–based assay (TaqMan; ABI, http://www.appliedbiosystems.com/) analyzed on an ABI Prism 7900 Real Time PCR System. Within each ethnic group, genotype completeness rates exceed 98% and there was no evidence for deviation from Hardy–Weinberg equilibrium (*p* > 0.05).

### Statistical analysis.

To ensure adequate control of type I error rates, we applied an inverse normal transformation to each trait prior to analysis [20]. The inverse normal transformation reduces the impact of outliers and deviations from normality on statistical analysis. The transformation involves ranking all available phenotypes, transforming these ranks into quantiles and, finally, converting the resulting quantiles into normal deviates. We included sex, age, and age^2^ as covariates in all analysis. Height was significantly associated with weight and hip circumference and was included as an additional covariate in analysis of those traits. We fitted a simple regression model to each trait and used a variance component approach to account for correlation between different observed phenotypes within each family. For individuals who had genotype data available, we coded genotypes as 0, 1, and 2 (depending on the number of copies of the allele being tested). For individuals with missing genotype data, we used the Lander–Green algorithm to estimate an expected genotype score (between 0 and 2) for each individual [24]. Briefly, to estimate each genotype score we first calculate the likelihood of the observed genotype data. Then, we instantiate each missing genotype to a specific value and update the pedigree likelihood. The ratio of the two likelihoods gives a posterior probability that the instantiated genotype is true, conditional on all available data. Due to computational constraints, we divided large pedigrees into subunits with “bit-complexity” of 19 or less (typically, 20–25 individuals) before estimating missing genotypes.

Our analytical approach considers all observed or estimated genotypes (rather than focusing on alleles transmitted from heterozygous parents) and thus is not immune to effects of population stratification. In homogenous populations, this type of analysis is expected to be more powerful [51,52]. To adjust for the effects of population structure and cryptic relatedness among sampled individuals, we used the genomic control method to adjust our test statistics for each trait separately [29]. FDRs were calculated with R's p.adjust() procedure using the method of Benjamini and Hochberg [30]. Since the initial analysis often identified clusters of nearby SNPs that all showed similar levels of association, we also carried out a sequential stepwise analysis. In this analysis, we selected the best SNP for each trait, and then conditioned on it to successively select the next best SNP. This sequential analysis can help identify regions with multiple independent association signals. The stepwise analysis was repeated for five rounds.

### Candidate gene analysis.

We selected 74 candidate genes previously tested for association with obesity in humans [53]. For each gene, we first evaluated the ability of the Affymetrix SNPs to tag common SNPs (MAF > 0.05) within +/− 5 kb of the gene (*r* ^2^ > 0.50 or *r* ^2^ > 0.80) using the HapMap CEU database [54]. We then evaluated evidence for association using all Affymetrix SNPs within each gene as well as neighboring Affymetrix SNPs that could be used to improve coverage (*r* ^2^ > 0.5). For each gene, we report coverage statistics as well as the SNP that showed strongest evidence for association.

We selected 74 genes that were previously targeted in associations studies aiming to identify genetic determinants of obesity in humans [53]: *ACE, ACTN, ADIPOQ, ADIPOR1, ADIPOR2, ADRB1, ADRB2, AGER, AHSG, APOA2, APOA4, APOA5, AR, BDNF, CASQ1, COL1A1, COMT, CRP, CYP11B2, DIO1, ENPP1, ESR1, ESR2, FABP2, FOXC2, GAD2, GFPT1, GHRHR, GNAS, GNB3, GPR40, H6PD, HSD11B1, HTR2C, ICAM1, IGF1, IGF2, IL6, IL6R, KCNJ11, KL, LEP, LEPR, LIPC, LPL, LTA, MC4R, MCHR1, MKKS, MTHFR, MTTP, NMB, NOS3, NPY, NPY2R, NR0B2, NTRK2, PARD6A, PLIN, PPARG, PPARGC1A, PRDM2, PTPN1, PYY, RETN, SCD, SELE, SERPINE1, TAS2R38, TNF, UCP1, UCP2, UCP3*, and *VDR*. We did not consider genes associated with drug-induced body weight gain or mitochondrial genes [53].

The following genes have previously been investigated for their role in obesity and related traits but are not well tagged by SNPs in the Affymetrix array: *ADRB3, DRD4, INS,* and *APOE*.

## Supporting Information

Table S1 Genotype Data for Sardinian Cohort (47 KB DOC)

Table S2 Results of Stepwise Analysis to Identify Independent Risk Alleles To generate this table, we first sought the most significantly associated allele in the genome. We then added this allele to our baseline model and repeated our genome scan to identify the next associated SNPs.
(55 KB DOC)

Table S3 Tag SNP That Shows Strongest Association with BMI for Each Previously Identified Candidate Gene The first column indicates the name of a previously identified candidate. The second column indicates the number of SNPs in our Affymetrix arrays that are either in the gene or constitute the best available tag (r^2^ > 0.5) for a genic SNP. The next column indicates the number of HapMap SNPs within +/− 5 kb of the gene and the proportion of these that are covered at *r* ^2^ > 0.50 or *r* ^2^ > 0.80. The next columns indicate the SNP that showed strongest association in our analysis, the *p*-value, the tested allele and its frequency, and the estimated additive effect. The last column corresponds to the FDR incurred when all tested SNPs are considered and this test is declared significant.
(162 KB DOC)

Table S4 Tag SNP That Shows Strongest Association with Hip Circumference for Each Previously Identified Candidate Gene The first column indicates the name of a previously identified candidate. The second column indicates the number of SNPs in our Affymetrix arrays that are either in the gene or constitute the best available tag (*r* ^2^ > 0.5) for a genic SNP. The next column indicates the number of HapMap SNPs within +/− 5 kb of the gene and the proportion of these that are covered at *r* ^2^ > 0.50 or *r* ^2^ > 0.80. The next columns indicate the SNP that showed strongest association in our analysis, the *p*-value, the tested allele and its frequency, and the estimated additive effect. The last column corresponds to the FDR incurred when all tested SNPs are considered and this test is declared significant.
(163 KB DOC)

Table S5 Tag SNP That Shows Strongest Association with Weight for Each Previously Identified Candidate Gene The first column indicates the name of a previously identified candidate. The second column indicates the number of SNPs in our Affymetrix arrays that are either in the gene or constitute the best available tag (*r* ^2^ > 0.5) for a genic SNP. The next column indicates the number of HapMap SNPs within +/− 5 kb of the gene and the proportion of these that are covered at *r* ^2^ > 0.50 or *r* ^2^ > 0.80. The next columns indicate the SNP that showed strongest association in our analysis, the *p*-value, the tested allele and its frequency, and the estimated additive effect. The last column corresponds to the FDR incurred when all tested SNPs are considered and this test is declared significant.
(163 KB DOC)

## Abbreviations

AA: African American
BMI: body mass index
CEU: Utah residents with ancestry from northern and western Europe
EA: European American
FDR: false-discovery rate
HA: Hispanic American
LD: linkage disequilibrium
YRI: Yoruba in Ibadan, Nigeria
