# Supplementary material for: Genome-Wide Association Scan Shows Genetic Variants in the FTO Gene Are Associated with Obesity-Related Traits
Source: PLoS Genet. 2007 Jul 20;3(7):e115. doi: 10.1371/journal.pgen.0030115 (PMC1934391; doi:10.1371/journal.pgen.0030115)
Supplement: Supplementary file 1 [file pgen.0030115.st001.doc]

**Supplementary Table 1 –Genotype Data for Sardinian Cohort**

|  | **10K** | **500K** | **Combined** |
| --- | --- | --- | --- |
| **# Markers** | 10,204 | 500,568 | 508,628 |
| **#Individuals** | 3,329 | 1,412 | 4,298 |
|  |  |  |  |
| **Spacing** |  |  |  |
| Mean (Median) | 287.6 Kb (141 Kb) | 5.8 Kb (2.5 Kb) | 5.7 Kb (2.4 Kb) |
| 0.80 percentile | 440Kb | 8.7 Kb | 8.5 Kb |
| 0.90 percentile | 998 Kb | 20.7 Kb | 14.1 Kb |
| Max | 28.7 Mb | 20.5 Mb | 20.5Mb |
|  |  |  |  |
| **Average heterozygosity** | 0.33 | 0.26 | 0.26 |
|  |  |  |  |
| **#Markers removed for:** |  |  |  |
| Call rate <90% | 181 | 3,975 |  |
| MAF <0.05 | 911 | 130,382 |  |
| HWE^1^ | 423 | 7,842 |  |
| Mendelian Inconsistency^2^ | 367 | 2,010 |  |
| Unlikely Genotypes^3^ | 720 | NA |  |
| Unknown position | 195 | NA |  |
|  |  |  |  |
| **#Markers that pass QC** | 7,407 | 356,359 | 362,129 |
|  |  |  |  |
| **Avg. genotypes per person** | 10,103.90 (99.0%) | 495,030.40 (98.8%) |  |
| **Avg. QC+ genotypes per person** | 7,289.35 (98.4%) | 353,007.30 (99.0%) |  |

^1^ Markers that fail an exact HWE test with a threshold of p < .001 (10K) and p < .000001 (500K)

^2^ Markers with Mendelian inconsistencies in >4 trios (of 1266 genotyped trios, 10K) and >2 trios (of 67 trios, 500K)

^3^Markers were Merlin's error detection flagged unlikely genotypes in >4 families.
