# Supplementary material for: Genome-Wide Association Scan Shows Genetic Variants in the FTO Gene Are Associated with Obesity-Related Traits
Source: PLoS Genet. 2007 Jul 20;3(7):e115. doi: 10.1371/journal.pgen.0030115 (PMC1934391; doi:10.1371/journal.pgen.0030115)
Supplement: Supplementary file 2 [file pgen.0030115.st002.doc]

**Supplementary Table 2 – Results of Stepwise Analysis, to identify independent risk alleles.**

To generate this table, we first sought the most significantly associated allele in the genome. We then added this allele to our baseline model and repeated our genome scan to identify the next associated SNPs.

| **Trait** | **SNP** | **Chr** | **Position** | **allele** | **freq** | **effect** | **H2** | **p-value** |
| --- | --- | --- | --- | --- | --- | --- | --- | --- |
|  |  |  |  |  |  |  |  |  |
| BMI | rs9930506 | 16 | 52387966 | A | 0.54 | -0.132 | 1.34% | 8.6E-07 |
|  | rs6602024 | 10 | 3145237 | G | 0.88 | -0.191 | 1.21% | 7.4E-06 |
|  | rs5922023 | X | 83320299 | C | 0.53 | 0.086 | 0.58% | 2.2E-05 |
|  | rs658838 | 6 | 128062178 | A | 0.59 | 0.095 | 0.69% | 5.9E-06 |
|  | SNP_A-2284869 | 5 | 162186248 | T | 0.85 | -0.148 | 0.90% | 2.2E-05 |
|  |  |  |  |  |  |  |  |  |
| HIP | rs9930506 | 16 | 52387966 | A | 0.54 | -0.157 | 1.63% | 3.4E-08 |
|  | rs2180579 | 20 | 621748 | C | 0.89 | 0.215 | 1.18% | 8.3E-06 |
|  | rs965670 | 8 | 120666727 | C | 0.95 | 0.211 | 0.62% | 9.1E-06 |
|  | rs1188445 | 1 | 30832969 | G | 0.91 | -0.208 | 0.92% | 1.9E-05 |
|  | rs1196845 | 12 | 104001507 | A | 0.68 | -0.131 | 1.01% | 2.1E-05 |
|  |  |  |  |  |  |  |  |  |
| WEIGHT | rs9930506 | 16 | 52387966 | A | 0.54 | -0.118 | 1.34% | 9.1E-07 |
|  | rs658838 | 6 | 128062178 | A | 0.59 | -0.086 | 0.69% | 6.8E-06 |
|  | SNP_A-2284869 | 5 | 162186248 | T | 0.85 | -0.141 | 0.99% | 9.5E-06 |
|  | rs7430259 | 3 | 74646178 | T | 0.85 | -0.136 | 0.91% | 2.2E-05 |
|  | rs6965526 | 7 | 85760188 | A | 0.65 | -0.099 | 0.89% | 3.3E-05 |
|  |  |  |  |  |  |  |  |  |
