# Supplementary material for: Genome-Wide Association Scan Shows Genetic Variants in the FTO Gene Are Associated with Obesity-Related Traits
Source: PLoS Genet. 2007 Jul 20;3(7):e115. doi: 10.1371/journal.pgen.0030115 (PMC1934391; doi:10.1371/journal.pgen.0030115)
Supplement: Supplementary file 5 [file pgen.0030115.st005.doc]

**Supplementary Table 5 – SNP tagging previously identified candidate gene showing strongest association with Weight.**

The first column indicates the name of a previously identified candidate. The second column indicates the number of SNPs in our Affymetrix arrays that are either in the gene or constitute the best available tag (r^2^ > 0.5) for a genic SNP. The next column indicates the number of HapMap SNPs within +/- 5kb of the gene and the proportion of these that are covered at r^2^ > 0.50 or r^2^ > 0.80. The next columns indicate the SNP that showed strongest association in our analysis, the p-value, the tested allele and its frequency, and the estimated additive effect. The last column corresponds to the FDR incurred when all tested SNPs are considered and this test is declared significant.

| **GENE** | **AffyX**  **SNPs** | **HapMap**  **SNPs** | **Coverage**  **(r2 >0.5)** | **Coverage**  **(r2>0.8)** | **SNP** | **p-value** | **allele** | **freq** | **effect** | **FDR** |
| --- | --- | --- | --- | --- | --- | --- | --- | --- | --- | --- |
| PRDM2 | 37 | 124 | 0.823 | 0.718 | rs1203628 | 0.0043 | T | 0.9 | 0.113 | 0.86 |
| ADIPOR2 | 17 | 71 | 0.972 | 0.915 | rs2286385 | 0.0134 | G | 0.57 | -0.063 | 0.86 |
| AHSG | 7 | 35 | 0.743 | 0.714 | rs2070635 | 0.0146 | G | 0.58 | -0.059 | 0.86 |
| ADIPOR1 | 4 | 26 | 0.769 | 0.462 | rs3820152 | 0.0159 | A | 0.56 | 0.061 | 0.86 |
| LPL | 11 | 59 | 0.729 | 0.576 | rs17410783 | 0.0172 | T | 0.82 | -0.076 | 0.86 |
| PPARGC1A | 14 | 109 | 0.624 | 0.459 | rs10938963 | 0.0199 | G | 0.56 | 0.057 | 0.86 |
| ADRB2 | 11 | 26 | 0.692 | 0.538 | rs877743 | 0.0205 | A | 0.85 | -0.078 | 0.86 |
| LEPR | 46 | 219 | 0.95 | 0.863 | rs12405556 | 0.0232 | G | 0.81 | 0.067 | 0.86 |
| UCP1 | 15 | 29 | 0.966 | 0.897 | rs2036020 | 0.0313 | G | 0.72 | -0.055 | 0.86 |
| FABP2 | 13 | 55 | 0.964 | 0.782 | rs6841872 | 0.0326 | G | 0.89 | -0.083 | 0.86 |
| GPR40 | 2 | 20 | 0.25 | 0.1 | rs12975589 | 0.0326 | C | 0.67 | -0.053 | 0.86 |
| GAD2 | 15 | 98 | 0.939 | 0.837 | rs3781108 | 0.0333 | T | 0.85 | -0.071 | 0.86 |
| SELE | 24 | 57 | 0.982 | 0.93 | rs12127655 | 0.0354 | C | 0.82 | 0.065 | 0.86 |
| NTRK2 | 72 | 337 | 0.846 | 0.694 | rs1539676 | 0.0359 | C | 0.71 | 0.057 | 0.86 |
| CRP | 6 | 19 | 0.947 | 0.842 | rs876538 | 0.0424 | C | 0.87 | -0.073 | 0.86 |
| LIPC | 41 | 202 | 0.683 | 0.475 | rs17190566 | 0.0446 | A | 0.84 | 0.064 | 0.86 |
| RETN | 2 | 8 | 0.375 | 0.375 | rs12610253 | 0.0466 | C | 0.8 | 0.065 | 0.86 |
| CASQ1 | 9 | 16 | 0.938 | 0.75 | rs6697826 | 0.0561 | T | 0.86 | -0.066 | 0.864 |
| PYY | 6 | 28 | 0.607 | 0.571 | rs1642598 | 0.0582 | A | 0.64 | -0.048 | 0.864 |
| H6PD | 8 | 32 | 0.594 | 0.469 | rs10489436 | 0.0597 | A | 0.87 | 0.068 | 0.864 |
| MCHR1 | 6 | 14 | 0.714 | 0.286 | rs5758108 | 0.0606 | G | 0.9 | -0.077 | 0.864 |
| HSD11B1 | 13 | 40 | 0.775 | 0.65 | rs11580294 | 0.0658 | G | 0.82 | 0.057 | 0.864 |
| FOXC2 | 2 | 6 | 0.167 | 0 | rs7204395 | 0.0668 | T | 0.93 | 0.089 | 0.864 |
| ACTN3 | 8 | 22 | 1 | 0.773 | rs509556 | 0.0742 | G | 0.53 | 0.046 | 0.864 |
| PPARG | 22 | 120 | 0.8 | 0.7 | rs6785890 | 0.0765 | C | 0.78 | 0.051 | 0.864 |
| GNAS | 10 | 43 | 0.628 | 0.419 | rs12625436 | 0.0782 | G | 0.62 | 0.046 | 0.864 |
| NR0B2 | 4 | 9 | 1 | 0.667 | rs11581460 | 0.083 | G | 0.72 | -0.046 | 0.864 |
| GHRHR | 6 | 26 | 0.5 | 0.231 | rs4723038 | 0.0902 | G | 0.91 | -0.07 | 0.884 |
| NPY2R | 9 | 25 | 0.76 | 0.4 | rs2342653 | 0.0908 | G | 0.56 | -0.043 | 0.884 |
| IL6 | 12 | 32 | 0.812 | 0.688 | rs2069835 | 0.0985 | T | 0.88 | -0.06 | 0.893 |
| SERPINE1 | 5 | 20 | 0.55 | 0.45 | rs6954408 | 0.1011 | C | 0.81 | 0.052 | 0.893 |
| COL1A1 | 5 | 22 | 0.682 | 0.455 | rs7406586 | 0.1144 | G | 0.78 | -0.044 | 0.921 |
| APOA5 | 6 | 12 | 0.917 | 0.5 | rs11600380 | 0.116 | T | 0.93 | -0.073 | 0.924 |
| HTR2C | 26 | 204 | 0.995 | 0.956 | rs950463 | 0.1238 | C | 0.87 | 0.034 | 0.95 |
| MKKS | 17 | 60 | 0.9 | 0.833 | rs6514111 | 0.1281 | A | 0.92 | -0.066 | 0.957 |
| VDR | 22 | 76 | 0.684 | 0.539 | rs2239182 | 0.1303 | T | 0.51 | -0.037 | 0.957 |
| ESR1 | 42 | 272 | 0.779 | 0.614 | rs1884054 | 0.1399 | A | 0.72 | -0.04 | 0.966 |
| ICAM1 | 1 | 16 | 0.438 | 0.438 | rs2569702 | 0.1399 | T | 0.57 | 0.037 | 0.966 |
| COMT | 9 | 44 | 0.75 | 0.5 | rs4646312 | 0.1431 | T | 0.51 | 0.037 | 0.966 |
| ADIPOQ | 8 | 29 | 0.586 | 0.483 | rs3774261 | 0.1484 | A | 0.54 | -0.036 | 0.986 |
| KL | 24 | 96 | 0.948 | 0.844 | rs659117 | 0.1579 | G | 0.85 | -0.047 | 0.991 |
| BDNF | 13 | 45 | 0.911 | 0.867 | rs12288512 | 0.16 | G | 0.9 | -0.054 | 0.991 |
| IGF1 | 19 | 49 | 0.857 | 0.776 | rs35765 | 0.1663 | G | 0.9 | -0.062 | 0.991 |
| LEP | 6 | 38 | 0.553 | 0.289 | rs4731429 | 0.1695 | G | 0.59 | -0.034 | 0.991 |
| ADRB1 | 1 | 10 | 0.6 | 0.6 | rs7090339 | 0.1789 | A | 0.87 | 0.047 | 0.991 |
| UCP2 | 2 | 17 | 0.706 | 0.588 | rs17132534 | 0.2028 | T | 0.92 | -0.055 | 0.991 |
| DIO1 | 11 | 25 | 0.92 | 0.76 | rs731828 | 0.207 | A | 0.7 | 0.033 | 0.991 |
| ENPP1 | 12 | 89 | 0.573 | 0.404 | rs7767502 | 0.207 | G | 0.81 | -0.049 | 0.991 |
| UCP3 | 5 | 24 | 0.958 | 0.583 | rs11235972 | 0.2399 | G | 0.87 | 0.041 | 0.991 |
| IGF2 | 4 | 12 | 0.833 | 0.5 | rs6578985 | 0.2429 | G | 0.66 | -0.031 | 0.991 |
| CYP11B2 | 8 | 29 | 0.966 | 0.897 | rs4543 | 0.2572 | C | 0.86 | -0.042 | 0.991 |
| AGER | 9 | 28 | 0.429 | 0.179 | rs204991 | 0.2805 | T | 0.93 | -0.065 | 0.991 |
| ESR2 | 10 | 78 | 0.628 | 0.474 | rs7154455 | 0.3088 | G | 0.62 | 0.025 | 0.991 |
| IL6R | 11 | 40 | 0.925 | 0.7 | rs2054855 | 0.3108 | C | 0.85 | -0.033 | 0.991 |
| LTA | 2 | 1 | 1 | 0 | rs1799964 | 0.3458 | T | 0.58 | 0.029 | 0.991 |
| TNF | 1 | 3 | 0 | 0 | rs1799964 | 0.3458 | T | 0.58 | 0.029 | 0.991 |
| PTPN1 | 9 | 60 | 0.85 | 0.733 | rs13045716 | 0.3488 | C | 0.64 | -0.023 | 0.991 |
| SCD | 6 | 31 | 0.742 | 0.581 | rs3793766 | 0.3508 | C | 0.9 | 0.037 | 0.991 |
| ACE | 7 | 30 | 0.933 | 0.66 | rs12601211 | 0.3537 | G | 0.58 | -0.023 | 0.991 |
| MTHFR | 9 | 50 | 0.88 | 0.54 | rs9651118 | 0.3577 | T | 0.93 | -0.046 | 0.991 |
| PLIN | 4 | 11 | 0.636 | 0.273 | rs1540504 | 0.3667 | C | 0.88 | 0.033 | 0.991 |
| APOA2 | 6 | 23 | 0.522 | 0.261 | rs4489574 | 0.3875 | C | 0.69 | -0.023 | 0.991 |
| KCNJ11 | 7 | 17 | 0.706 | 0.529 | rs7124355 | 0.3885 | G | 0.63 | 0.021 | 0.991 |
| GFPT1 | 12 | 37 | 0.919 | 0.919 | rs4302256 | 0.3924 | A | 0.59 | -0.022 | 0.991 |
| MTTP | 13 | 46 | 0.913 | 0.826 | rs11935423 | 0.4092 | C | 0.88 | 0.029 | 0.991 |
| PARD6A | 2 | 2 | 1 | 1 | rs7198357 | 0.5101 | C | 0.78 | -0.019 | 0.991 |
| TAS2R38 | 1 | 4 | 0.25 | 0 | rs9640357 | 0.549 | C | 0.74 | -0.017 | 0.991 |
| APOA4 | 8 | 2 | 0.5 | 0 | rs7396835 | 0.5993 | G | 0.82 | 0.017 | 0.991 |
| NPY | 7 | 40 | 0.75 | 0.55 | rs16082 | 0.608 | A | 0.84 | -0.016 | 0.991 |
| NMB | 7 | 19 | 0.789 | 0.737 | rs1107179 | 0.6109 | G | 0.59 | -0.013 | 0.991 |
| AR | 2 | 26 | 0.923 | 0.692 | rs4827545 | 0.6792 | G | 0.85 | -0.011 | 0.991 |
| GNB3 | 1 | 17 | 0.176 | 0.059 | rs10849538 | 0.7022 | A | 0.78 | -0.011 | 0.991 |
| MC4R | 4 | 9 | 0.889 | 0.333 | rs17700926 | 0.8466 | A | 0.88 | -0.007 | 0.991 |
| NOS3 | 1 | 14 | 0.071 | 0.071 | rs3918188 | 0.8743 | C | 0.7 | 0.004 | 0.991 |
